# The Role of Transient Receptor Potential Vanilloid 1 in Common Diseases of the Digestive Tract and the Cardiovascular and Respiratory System

**DOI:** 10.3389/fphys.2019.01064

**Published:** 2019-08-21

**Authors:** Qian Du, Qiushi Liao, Changmei Chen, Xiaoxu Yang, Rui Xie, Jingyu Xu

**Affiliations:** Department of Gastroenterology, Affiliated Hospital to Zunyi Medical University, Zunyi, China

**Keywords:** transient receptor potential vanilloid 1, digestive tract, cardiovascular system, respiratory system, functional dyspepsia, irritable bowel syndrome

## Abstract

Transient receptor potential vanilloid subtype 1 (TRPV1), a member of the transient receptor potential vanilloid (TRPV) channel family, is a nonselective cation channel that is widely expressed in sensory nerve fibers and nonneuronal cells, including certain vascular endothelial cells and smooth muscle cells. The activation of TRPV1 may be involved in the regulation of various physiological functions, such as the release of inflammatory mediators in the body, gastrointestinal motility function, and temperature regulation. In recent years, a large number of studies have revealed that TRPV1 plays an important role in the physiological and pathological conditions of the digestive system, cardiovascular system, and respiratory system, but there is no systematic report on TRPV1. The objective of this review is to explain the function and effects of TRPV1 on specific diseases, such as irritable bowel syndrome, hypertension, and asthma, and to further investigate the intrinsic relationship between the expression and function of TRPV1 in those diseases to find new therapeutic targets for the cure of related diseases.

## Introduction

The transient receptor potential vanilloid (TRPV) subfamily consists of six members and is divided into four groups based on homology: TRPV1/TRPV2, TRPV3, TRPV4, and TRPV5/TRPV6. Members of the TRPV subfamily function as tetrameric complexes with each subunit containing six N-terminal ankyrin repeats. All members have a TRP box in their C-terminus. TRPV1, TRPV2, TRPV3, and TRPV4 are modestly permeable to Ca^2+^, whereas TRPV5 and TRPV6 are the only channels that are highly selective for Ca^2+^ (Nilius and Owsianik, 2011). Although most members of the TRPV family are modestly permeable to Ca^2+^, they may play different roles in the body due to their differences in structure and distribution. TRPV1, the most widely studied member of the TRPV family, is a nonselective cationic ligand-gated channel located extensively on neuronal cells or nonneuronal cell membranes with high permeability to Ca^2+^. TRPV1 acts as a multisensory receptor for potential injury signals and can be activated by a variety of exogenous and endogenous mediators, such as capsaicin, temperature (43–52°C), acidic environments (H^+^), and leukotriene B4 (LTB4). The activation of TRPV1 primarily permits an influx of extracellular Ca^2+^, which is involved in a number of essential physiological functions, such as neurotransmitter release, membrane excitability, and muscle cell contraction (Jordt and Ehrlich, 2007). Conversely, TRPV2 cannot be activated by capsaicin, H^+^, or heat (<50°C), except for noxious heat (>53°C); despite this, TRPV2 shows a 50% sequence similarity to TRPV1. Moreover, TRPV2 is probably the least understood member of the TRPV subfamily, and the regulation of TRPV2 in humans is unknown (Caterina et al., 1997). Similar to TRPV2, TRPV3 does not respond to capsaicin, H^+^, or temperature below 50°C but is activated by innocuous temperatures above 30–33°C. TRPV3 is currently considered to be a nonselective cation channel activated by mild stimulation. TRPV3 is highly expressed in keratinocytes and is essential for several skin functions, including skin barrier formation and hair morphogenesis (Cheng et al., 2010). TRPV4 is activated by moderate temperatures (>24°C). Unlike TRPV1, TRPV4 is not activated by capsaicin but is sensitive to changes in extracellular osmotic pressure (Nilius and Owsianik, 2011). TRPV5 and TRPV6 are relatively unique subgroups within the family and exhibit low homology to TRPV 1. TRPV5 and TRPV6 are the only highly Ca^2+^-selective TRP channels and are mainly present in tissues and organs related to calcium ion transport. The regulation of TRPV1 and TRPV2 is related to the reabsorption of calcium by renal and intestinal epithelial cells. Current research has focused on the expression and regulation of TRPV5 and TRPV6 in the kidney and small intestine (Hoenderop et al., 2005). Compared to other TRPV channels, TRPV1 is the major channel for the detection and integration of nociceptive chemical and thermal stimuli in sensory nerve fibers (thin myelinated Ad-fibers and unmyelinated C fibers), innervating most of our organs. TRPV1 activation initiates inflammation and the transmission of pain signals. The rapid progress in TRPV1 channel research has provided greater understanding of the pathogenic roles that this channel plays in the development and maintenance of acquired diseases. Many studies have examined the TRPV1 mechanism, and drug discovery research on TPRV1 has been conducted in various disease fields. In this review, a brief summary is given on the progress of research on TRPV1 in the digestive system, cardiovascular system and respiratory system in recent years.

## Structure of TRPV1

**Figure 1:**
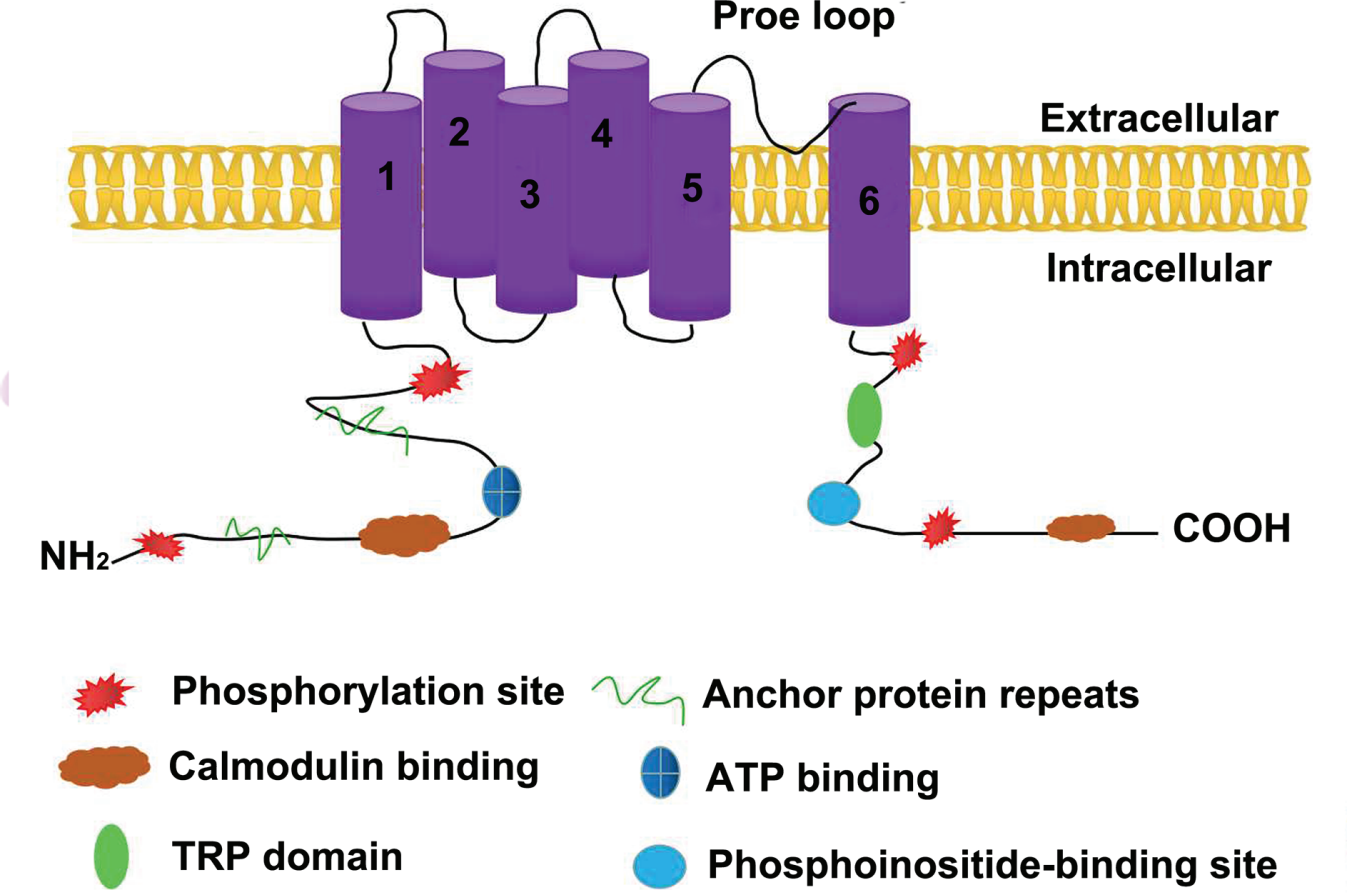
The structure of TRPV1. TRPV1 is a protein with six transmembrane regions, the fifth transmembrane protein region and the sixth transmembrane protein region of each subunit aggregate together to form a pore region for the TRPV1 channel. The amino-terminal and carboxy-terminal of TRPV1 are both intracellular.There are three anchor protein repeats and multiple phosphorylation binding sites at the N-terminus, which can bind to calmodulin and ATP. The N-terminal intracellular region contain TRP domain,calmodulin-binding sites and phosphoinositide-binding sites.

TRPV1 is the first member of the TRPV subfamily that was discovered and cloned. Caterina et al. (1997) successfully cloned a receptor that can be activated by capsaicin; thus, TRPV1 is also known as the capsaicin receptor. The cDNA of TRPV1 has an open reading frame of 2,514 bp and encodes a protein of 828 amino acids. It possesses a tetrameric structure consisting of six transmembrane regions and a hydrophobic group located between the fifth and sixth transmembrane regions. Both the N-terminus and the C-terminus are located inside the cell membrane, regulating the functional activity of the protein. There are three anchor protein repeats and multiple phosphorylation binding sites at the N-terminus, which can bind to calmodulin and ATP. The TRP domain near the C-terminus, including a calmodulin-binding region and a phosphoinositide-binding site, is involved in the regulation of voltage-gated channel opening and, combined with phosphoinositide and protein kinase, regulates the temperature sensor of the receptor. Moiseenkova-Bell et al. (2008) analyzed the structure of TRPV1 using cryo-electron microscopy at 19-Å resolution. The overall structure of TRPV1 was divided into upper and lower parts, corresponding to the transmembrane and intracellular regions of the channel. In December 2013, both the David Julius lab and the Yifan Cheng lab jointly reported the cryogenic structure of TRPV1 and obtained images of the protein using a direct electron detector. The structure shows that TRPV1 has length, width, and height dimensions of approximately 100 Å × 110 Å × 110 Å, respectively (Maofu et al., 2013). This study also reported a double-gated regulation mechanism for TRPV1. The fifth and sixth transmembrane protein regions of each subunit of TRPV1 aggregate together to form a pore region in the TRPV1 channel. The first to the fourth transmembrane proteins are symmetrically distributed in the four corners of the pore region (Figure 1). Capsaicin interacts with the cytoplasmic domain of the third and fourth transmembrane proteins on TRPV1 *via* hydrogen bonding. Ser512, the third transmembrane protein, plays an essential role in recognizing pH and capsaicin (Johnson et al., 2006). Fan et al. (2018) found that the TRPV1 channel closed when the temperature dropped to the freezing point even in the presence of capsaicin, and the fully open TRPV1 model combined with capsaicin was deduced through computer modeling analysis. This model demonstrated how the TRPV1 channel is activated from a closed state as well as the activation mechanism of capsaicin, which in turn can trigger corresponding biological effects and affect intestinal inflammation, metabolic syndrome, and other diseases (McCarty et al., 2015).

## Distribution and Biological Characteristics of TRPV1

**Figure 2:**
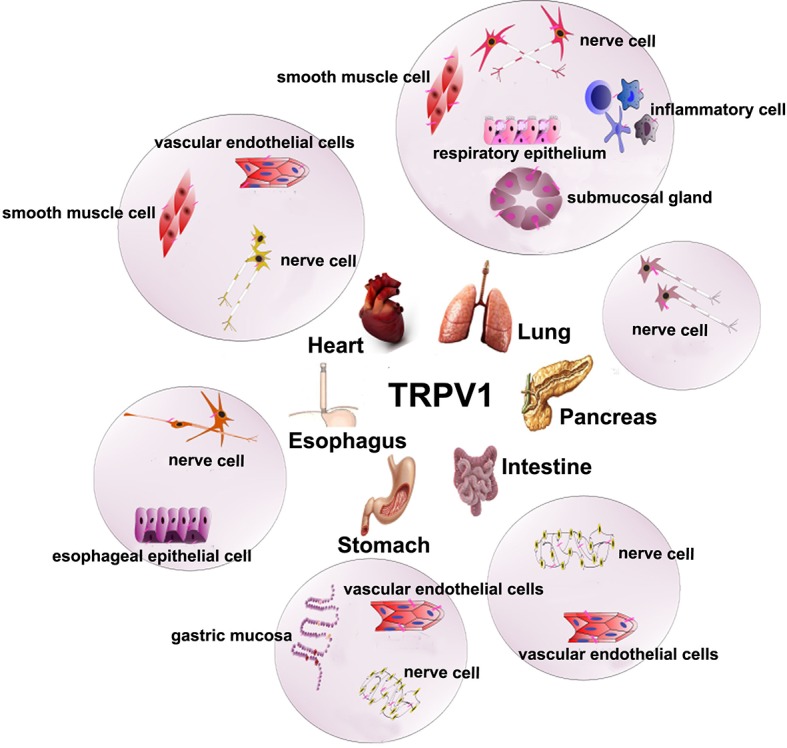
The expression of TRPV1 in digestion, cardiovascular, and respiratory system. TRPV1 is mainly expressed nerve cells around the esophageal mucosa and blood vessels, esophageal epithelial cells in the esophagus. The submucosal plexus, myenteric plexus, vascular endothelial cell, gastric parietal cells, gastric main cells, and gastric antral G cells in the stomach. The nerve cells in pancreas. The submucosal plexus, myenteric plexus, and vascular endothelial cells in the intestine.TRPV1 is distributed in vascular peripheral nerve cells, smooth muscle cells, vascular endothelial cells in cardiovascular. It is also expressed in sensory nerve fibers, smooth muscle cells, vascular endothelial cells, submucosal glands, and inflammatory cells in the respiratory system.

In mammals, TRPV1 is distributed extensively in the unmyelinated C-type sensory nerve fibers and partially in the less myelinated Aδ-type sensory nerve fibers (Alawi and Keeble, 2010). In the peripheral neuron system, it is primarily expressed in the dorsal root ganglion, trigeminal ganglion, vagal ganglion, and other small neurons, and in the central nervous system, it is expressed in the thalamus, striatum, amygdala, and other regions. The pancreas, liver, lung, heart, and other organs in the human body also express TRPV1 (Bollimuntha et al., 2011). In the gastrointestinal tract, TRPV1 is mainly distributed in the submucosal nerve plexus of the intestinal tract, myenteric nerve plexus, gastrointestinal muscle mucosa, and some nonnerve tissue distributions, such as gastric mucosal cells, gastric parietal cells, and gastric antral G cells (Akbar et al., 2010). TRPV1 is mainly distributed in vascular endothelial cells (Alawi and Keeble, 2010; Akbar et al., 2010; Bollimuntha et al., 2011), smooth muscle cells (Luo et al., 2011), and perivascular nerve cells (Peng and Li, 2010) in the cardiovascular system. TRPV1 can be found widely distributed in respiratory sensory nerve fibers in the respiratory system in mammals, especially C-like sensory nerve fibers, which are mostly distributed throughout the respiratory system, including the upper respiratory tract to the lower respiratory tract and to the pulmonary parenchyma thereafter. Furthermore, TRPV1 is expressed in the smooth muscle cells, the vascular endothelial cells, the submucosal gland, and inflammatory cells in the respiratory system (Figure 2; Watanabe et al., 2006).

**Figure 3:**
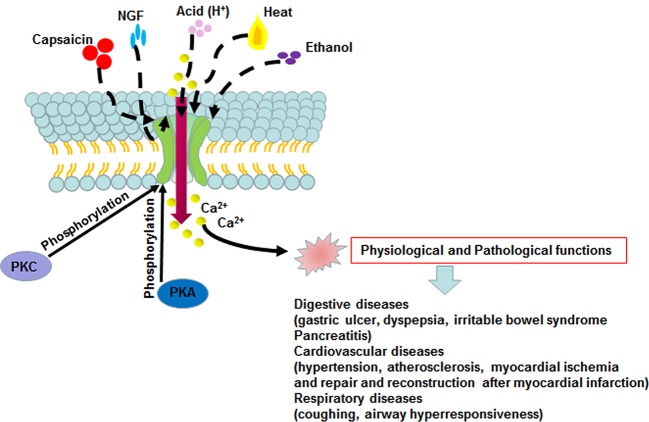
TRPV1 channel activation and its function in mammalian cell. The TRPV1 can be activated by capsaicin, heat, acidosis, NGF, ethanol phosphorylation PKA and PKC. The activation of TRPV1 induces an influx of extracellular Ca^2+^, resulting in increased intracellular calcium, and regulates the corresponding physiological and pathological functions.

TRPV1 is not only activated by capsaicin; it is also activated or sensitized directly or indirectly by a variety of physical and chemical factors and inflammatory mediators, such as mechanical stimulation, ethanol, inflammatory mediators, tissue damage, acid (pH less than 5.3), noxious heat stimulation (>43°C), alterations in extracellular osmotic pressure, an intracellular redox state, substance P (SP), nerve growth factor (NGF), and prostaglandin (Alawi and Keeble, 2010). The noxious signal generated by the activated TRPV1 can be blocked by a TRPV1 antagonist, and multiple molecules have been developed as TRPV1 antagonists, such as SB-705498 (Lambert et al., 2009), A99361 (Summ et al., 2011), etc. In addition, the desensitization strategy has been exploited in numerous TRPV1 therapeutics, including capsaicin cream (Zostrix), as well as high concentration patches (Qutenza) (Brederson et al., 2013). In recent years, some scholars have found that oxytocin, as an endogenous agonist, suppresses nociception and induces analgesia by specifically affecting inflammatory pain pathways in an animal model (Eliava et al., 2016), and this effect is mainly exerted by TRPV1 desensitization (Nersesyan et al., 2017). The activation of TRPV1 mainly permits an influx of extracellular Ca^2+^, resulting in increased intracellular calcium, which can depolarize the cells to produce action potentials and transmit nociceptive signals along the sensory nerve fibers to the nerve center or activate a series of signaling pathways in the cell, subsequently triggering a wide range of cellular responses. In addition, the increase in terminal calcium induced by activation of the TRPV1 receptor in sensory nerve fibers may allow for local vesicle release of neuropeptides, independent of action potential formation (Kaneko and Szallasi, 2014), to ultimately regulate the corresponding physiological and pathological functions. Results from existing studies have suggested that the following mechanisms mediate TRPV1 channel activation and thus regulate the body’s physiological function (Mitsuko et al., 2002; Stein et al., 2006; Vermeulen et al., 2013): (1) the threshold of TRPV1 can be lowered by NGF *via* activation of phospholipase C (PLC), which can induce the generation of phosphatidylinositol 3 kinase (PI3K) subunit p85 and the translocation of TRPV1 from the intracellular space to the cell membrane. This effect increases the level of TRPV1 expression on the cell membrane and later reduces the TRPV1 channel threshold, ultimately leading to TRPV1 sensitization (Stein et al., 2006). (2) Inhibition of TRPV1 by the phosphatidylinositol 4,5-diphosphate (PIP2) pathway is involved in channel sensitization to proinflammatory agents (Bhave and Th, 2010). (3) TRPV1 can be activated by phosphorylation protein kinase C (PKC), which is phosphorylated along the PLC-PKC pathway by tryptase and other substances (Mitsuko et al., 2002). (4) The channel can be sensitized by protein kinase A (PKA) pathway activation *via* monoamine neurotransmitters (Figure 3; Vermeulen et al., 2013).

## TRPV1 and Disease

### TRPV1 and Digestive Diseases

#### TRPV1 and Gastric Ulcer

Gastric ulcers are the most common type of peptic ulcer, which mainly refers to tissue damage beyond the mucosal muscle layer caused by gastric digestive juice. Traditionally, capsaicin has been considered to have a stimulating effect on the gastrointestinal tract. Oral administration of capsaicin to rats has been reported to cause acute erosive gastritis, which can be inhibited if a histamine H-2 receptor antagonist is administered in advance (Mann, 1977). For patients with gastric ulcers, it is generally recommended to either remove peppers from their diet or eat as little as possible. However, it has been reported that consuming average amounts of capsaicin has a protective effect on the gastric mucosa. Epidemiological studies have demonstrated that the incidence of gastric ulcers in a Singaporean Chinese population is three times lower than that in Indian and Malaysian populations, which is related to the fact that Indians and Malaysians eat more peppers (Kang et al., 1995). Szolcsányi and Barthó (1981) observed that, in an ulcer rat model, the formation of ulcers could be suppressed by injection of a low dose (10 or 50 g/ml) of capsaicin into the rats in advance. Another study further showed that capsaicin perfused into the stomach of rats can inhibit gastric mucosal injury, and the protective effect of capsaicin on gastric mucosa can be weakened after application of Nailhong and chili pepper, both of which are nonselective TRPV1 inhibitors (Horie et al., 2004). At present, the protective mechanism of capsaicin receptor channels on the gastric mucosa remains unclear and may be related to the following factors: (1) TRPV1 can regulate gastric acid secretion. A large amount of calcitonin gene-related peptides (CGRPs) is released after activation of TRPV1 in primary nociceptive nerves by small doses of capsaicin. CGRP has a strong inhibitory effect on irritation caused by gastric acid and pepsin secretion (Andrea et al., 2012). Mózsik et al. (2005) studied the effects of capsaicin on gastric mucosa and gastric acid secretion in 84 healthy subjects and found that the administration of low concentrations of capsaicin inhibited gastric acid secretion. Hirokuni et al. (2012) noted that the dietary TRPV1 agonists capsaicin and 6-gingerol can inhibit gastric acid secretion, and the combination of the two agonists can not only significantly reduce the secretion of gastric acid but also has an additive effect. (2) When TRPV1 is activated, nerve fibers can release substances such as tachykinins that promote gastric motility and accelerate gastric emptying (de Man et al., 2010). Gastric emptying was examined *via* the ^13^C-octanoic acid breath test after oral administration of capsaicin in healthy people, and the results showed that the gastric emptying time decreased from 112 ± 15 min to 99 ± 14 min, indicating that capsaicin can significantly accelerate the gastric emptying process (Debreceni et al., 1999). Yoshimura et al. (2016) found that the vagus nerve, which promotes the expression of TRPV1 in the inhibition of motilin by gastric acidification, has a considerable influence on the contractile activity of gastric acid regulating gastric motility. (3) TRPV1 can increase gastric mucosal blood flow. Capsaicin can protect the gastric mucosa by increasing the blood flow of gastric mucosa in rats. A previous study showed that capsaicin itself does not stimulate gastric acid secretion but rather inhibits it; it was also found to stimulate the secretion of alkaline mucus and to increase blood flow to the gastric mucosa, thus helping to prevent and cure ulcers (Satyanarayana, 2006). (4) TRPV1 can promote the secretion of prostaglandins and epidermal growth factor. Endogenous prostaglandin E2 is produced when the gastric mucosa is stimulated by small doses of capsaicin, and this molecule participates in mucosal protection. Epidermal growth factor (EGF), which promotes mucosal growth, is secreted when salivary glands are stimulated by capsaicin in serum (Arai et al., 2003).

However, TRPV1 activation in the sensory nerves of the stomach plays an important role in acute ethanol-mediated gastric mucosal injury. Activating TRPV1 causes the release of SP, which activates neurokinin type 1 receptor (NK1) in gastric epithelial cells, thereby increasing the generation of reactive oxygen species (ROS), the main cytotoxic agent of the gastric mucosa. The production of ROS leads to lipid peroxidation in the cell membrane, and these increased amounts of ROS, together with glutathione depletion and antioxidant enzyme inhibition, which reduce antioxidant defenses, cause gastric mucosal damage (Gazzieri et al., 2007). Moreover, the TRPV1 channel in rodents is a major target for the antinociceptive effect of the probiotic Lactobacillus reuteri DSM 17938 (Perez-Burgos et al., 2015). DSM17938 may decrease TRPV1 receptor expression, inhibiting the effects of ethanol-mediated TRPV1, thereby decreasing ROS levels in the gastric mucosa (Oliveira et al., 2019).

#### The Relationship of TRPV1 With Functional Dyspepsia and Irritable Bowel Syndrome

Functional dyspepsia (FD) refers to a group of clinical syndromes caused by gastric and duodenal dysfunction, with visceral hypersensitivity being a representative mechanism in its development. In 54 patients with FD, the score of dyspeptic symptoms was significantly increased after taking capsaicin capsules, suggesting that the capsaicin receptor channel is associated with functional dyspepsia (Hammer et al., 2010). At present, the mechanism by which TRPV1 leads to increased visceral sensitivity in patients with FD may be related to the following factors: (1) dephosphorylation of TRPV1 can desensitize visceral sensation, and it can restore sensitivity to capsaicin when TRPV1 is re-phosphorylated (Mandadi et al., 2004). (2) Capsaicin can bind with high affinity to specific compounds that contain SP on the membrane of the primary sensory nerve terminal (He-Bin et al., 2008). (3) CGRP is an inflammatory and pain-inducing primary afferent neurotransmitter that is found in capsaicin-sensitive afferent nerve fibers. The release of CGRP after TRPV1 activation is involved in the regulation of pain sensation (Andrea et al., 2012). (4) Proteolytic enzyme-activated receptor 2 (PAR2) has a modulatory effect on TRPV1 and can increase the sensitivity of cells to TRPV1 agonists (Vergnolle, 2009). Intriguingly, repeated ingestion of capsaicin over a prolonged period reduces the symptoms of FD but initially induces upper abdominal symptoms. Sensitizing chemonociception might be the cause for the initial effects of capsaicin. Symptom reduction after prolonged treatment with capsaicin in dyspeptic patients might be related to the desensitization of the TRPV 1 receptor to capsaicin. Not only the receptor but also the whole nerve fiber that subserves the receptor could become refractory to other nociceptive and mechanical stimuli. Thus, this effect provides a broader modulation of painful and burning symptoms than the effects of specific capsaicin antagonists that inhibit only a specific group of receptors. Therefore, the use of chili, a natural capsaicin agonist, to provide relief to patients with chronic gastrointestinal symptoms may provide a broader effect than specific capsaicin receptor antagonists, which may cause severe side effects by modifying gut symptoms (Fuhrer and Hammer, 2009).

Irritable bowel syndrome (IBS) is a functional bowel disorder that presents with no structural and biochemical abnormalities, and it is clinically characterized by abdominal pain, abdominal distention, alterations in bowel habits, and changes in stool characteristics. The present pathogenesis of IBS remains unclear. Related literature shows that visceral hypersensitivity, resulting from increased signal transmission between dorsal horn neurons and the brain, is a key factor underlying the generation of pain (Shi et al., 2013). The results of that study revealed that the expression of TRPV1 on nerve fibers was significantly upregulated in the colon tissue of patients with IBS, and this upregulation was positively correlated with the severity of abdominal pain. When TRPV1 is phosphorylated after ligand binding, the channel is opened, and cations (mainly Na^+^ and Ca^2+^) enter the intracellular space, mediating colonic smooth muscle contraction and promoting the release of neuropeptides and excitatory amino acids such as SP, CGRP, and other gastrointestinal peptides, which ultimately leads to increased visceral sensitivity and gastrointestinal dysfunction, resulting in IBS-related symptoms (Akbar et al., 2010). In contrast, TRPV1 knockout in mice did not result in heat or tactile hyperalgesia, and the sensitivity of colonic expansion stimulation was reduced by approximately 50% (Vardanyan et al., 2009). Pei et al. (2018) also confirmed that acupuncture stimulation can treat IBS by reducing TRPV1 activation by upregulating microRNA-199 expression in colon cells. The current research confirms that the following pathways may be involved in the hypersensitization of TRPV1 to promote the occurrence of IBS: (1) TRPV1 is activated by the PLC/PKC signaling pathway, which mediates the activation of histamine 1 receptor (HRH1) by histamine or histamine metabolites and mediates activation (Premkumar and Ahern, 2000). PLC indirectly sensitizes TRPV1 by chelation with PIP2, which can bind to TRPV1 (Prescott and David, 2003). PKγ in the PKC family plays an important role in the regulation of pain (Jianhua et al., 2015). It has been confirmed in the literature that the PKCγ⁃TRPV1⁃CGRP signaling pathway is vital for the activation, transduction, and regulation of IBS visceral pain signals (El-Salhy, 2015). Previous studies have shown that histamine and serotonin, which are released from the mast cells of the intestine, can also cause visceral hypersensitivity in mice (Cenac et al., 2010). The release of histamine and tryptase after mast cell activation was found to be significantly increased in IBS patients (Cenac et al., 2007). The released tryptase is cleaved and binds a G protein-coupled receptor PAR2 activating phosphatase C (PLC), which can decompose PIP2 on the membrane into diacylglycerol (DAG) and 1,4,5-inositol triphosphate (IP3). The intracellular release of Ca^2+^ after IP3 activation binds to calmodulin in the cytoplasm, while DAG, with the synergy of calcium ions, can activate PKC and thus mediate TRPV1 phosphorylation (Amadesi et al., 2010; Wickley et al., 2010). The influx of Ca^2+^ increases when the opening threshold of TRPV1 is decreased, leading to the abnormal perception of visceral pain and eventually leading to the occurrence of visceral hypersensitivity. Wouters et al. (2016) also confirmed that histamine and its metabolites can hypersensitize TRPV1 by activating HRH1 in both clinical trials and in mice. (2) Monoamine neurotransmitters such as dopamine and norepinephrine can be activated by G protein-coupled receptors, which then activate adenosine monophosphate (cAMP) and PKA *via* adenylate cyclase (AC) (Vermeulen et al., 2013). TRPV1 has a PKA regulatory domain, a binding site for cofactors such as cAMP, Ca^2+^, etc., which regulates enzyme activity by the binding or dissociation of these cofactors (Rathee et al., 2002). When PKA promotes the phosphorylation of TRPV1, the channel is open, and Ca^2+^ flows into the intracellular space, mediating colonic smooth muscle contraction and promoting the release of neuropeptides and excitatory amino acids such as SP, CGRP, and other gastrointestinal peptides, which ultimately leads to increased visceral sensitivity and gastrointestinal dysfunction, resulting in IBS-related symptoms (Hughes et al., 2009). (3) NGF is involved in enhancing the expression of TRPV1. In a recent study, bile acid (BA) treatment enhanced the protein expression of TRPV1 in the dorsal root ganglion (DRG), resulting in visceral hypersensitivity (VH). In this study, it was found to increase colonic BA and induce the expression and release of NGF in mast cells, which involves the farnesoid X receptor (FXR)/MKK3/6/p38MAPK/NF-kB signaling pathway. BA-stimulated, MMC-derived NGF mainly acts on the TrkA/TRPV1 axis in DRG neurons. BAs induce VH *via* mucosal mast cell-to-nociceptor signaling, which involves the farnesoid X receptor-nerve growth factor-transient receptor potential vanilloid 1 axis (Li et al., 2019).

As with FD, the desensitization effects of capsaicin ingestion in IBS patients were demonstrated in two randomized controlled trials (Aniwan and Gonlachanvit, 2014). For instance, the ingestion of chili for 6 weeks (capsaicin 2 mg/day) significantly reduced the abdominal pain and bloating induced by spicy meals compared with placebo (Aniwan and Gonlachanvit, 2014). There has been mounting evidence to support the role of the desensitization of capsaicin receptors in functional gastrointestinal disorders, including FD and IBS. These results also support the use of capsaicin, a natural capsaicin receptor agonist, for the treatment of IBS symptoms. However, further research is still needed to confirm this hypothesis. The results of studies on ghrelin, a brain-gut hormone, indicated that ghrelin exerted an antinociceptive effect that was mediated *via* TRPV1/opioid systems in IBS-induced visceral hypersensitivity (Mao et al., 2017). Ghrelin has been reported to show antinociceptive effects in peripheral pain. Moreover, TRPV1, κ opioid receptor (KOR) and μ opioid receptor (MOR) are coexpressed in the DRG, cerebral cortex, and colon tissue in a rat model of IBS. Compared with MD (maternal deprivation, a way to establish a stress-induced model of IBS in Wistar rats), the protein and mRNA levels of the opioid receptors KOR and MOR were significantly increased in the ghrelin-treated group, accompanied by a marked reduction in TRPV1 expression. These results suggested that ghrelin upregulates the expression of opioid receptors and subsequently inhibits TRPV1 activation, exerting an antinociceptive effect in IBS-induced VH. These findings indicate that ghrelin could be used as a new treatment for IBS.

Thus far, we know that the expression of TRPV1 in colonic nerve fibers is upregulated in patients with IBS and in patients with inflammatory bowel disease (IBD) (Akbar et al., 2010). Moreover, studies have shown the expression of TRPV1 in intestinal epithelial cells in patients with IBD, but the results regarding the role of TRPV1 in patients with IBD are conflicting. In a study of the expression of TRPV1 in colonic epithelium and its correlation with IBD, Chengxin Luo et al. studied 60 patients from China with active IBD, including 30 patients with ulcerative colitis (UC) and 30 patients with Crohn’s disease (CD). Their results showed that TRPV1 expression was significantly upregulated in the colonic epithelium of IBD patients compared with controls (Chengxin Luo et al., 2017). However, Rizopoulos et al. revealed that the expression of TRPV1 in the intestinal epithelium was reduced in 52 Greek patients (Rizopoulos et al., 2018). Because there is currently little research on TRPV1 in epithelial cells of IBD patients, more research is still needed to confirm the specific role of TRPV1 in IBD to provide a theoretical basis for the use of TRPV1 therapeutic targets in the treatment of IBD.

#### TRPV1 and Pancreatitis

The pancreas is an organ that has both exocrine and endocrine functions. The normal synthesis, storage, and secretion of digestive enzymes of the pancreatic exocrine glands are essential for maintaining the steady state of the pancreas and exerting its normal physiological functions. Pancreatic injury often initiates conversion of the proenzyme trypsinogen to its active form trypsin. When trypsin exceeds the capacity that endogenous trypsin inhibitors can handle, activation of other zymogens occurs within the pancreas, producing a proteolytic cascade that ultimately causes autodigestion of the gland. Pancreatitis occurs when this destructive process is secondarily accompanied by inflammatory features including cytokine release, vasodilation, edema, and neutrophil infiltration (Steer and Meldolesi, 1988). The neural innervation of the pancreas may play a very important role in initiating and maintaining the inflammatory response caused by pancreatic injury. The pancreas is dominated by sympathetic, parasympathetic neurons, and sensory nerve fibers that modulate the exocrine and endocrine functions of the pancreas. In the activation of pancreatic primary sensory neurons, which contain a number of bioactive transmitters—some of which cause local inflammation—two subpopulations of unmyelinated neurons, known as C and Aδ fibers, are particularly relevant. These fibers contain the tachykinins neurokinin A, neurokinin B, and SP as well as CGRP, glutamate, and adenosine, all of which have proinflammatory actions, resulting in the release of inflammatory neuropeptides to signal pain, and the pancreas itself causes neutrophil infiltration and plasma extravasation and the release of cytokines under noxious stimulation. Some studies have confirmed that SP and CGRP are associated with neurogenic inflammation and that they have the ability to interact with endothelial cells, arterioles, mast cells, and other immune cells to induce vasodilation, edema, and inflammatory cell infiltration (Mantyh et al., 1995). Wick et al. (2006) found that TRPV1 is expressed in nerve fibers close to the pancreatic acinus. Xu et al. (2007) found that the expression and function of TRPV1 were upregulated in a model of induced chronic pancreatitis in rats, and administration of TRPV1 antagonist significantly reduced visceral pain behavior and somatic hyperalgesia in rats with chronic pancreatitis. Previous studies have demonstrated that inflammatory damage can lead to the release of endogenous TRPV1 agonists in the pancreas, thereby activating TRPV1 in the primary sensory nerve, which in turn promotes the release of inflammatory neurotransmitters such as SP and CGRP. The above results indicate that TRPV1 receptor activation is associated with the production of pancreatic inflammation and pain (Shahid et al., 2015).

Acute pancreatitis (AP) is defined as an inflammatory event with self-limiting characteristics. Although abdominal pain is a common symptom of AP, little is known about the mechanism by which pancreatic inflammation activates sensory nerves and then causes pain. Some researchers have confirmed that nerves expressing TRPV1 are involved in neurogenic inflammation during acute pancreatitis (Romac et al., 2008). Nathan et al. (2001) demonstrated that treatment with the TRPV1 antagonist capsazepine significantly reduced the severity of pancreatitis in experimental pancreatitis caused by administration of caerulein, suggesting that TRPV1 mediates the inflammatory process of pancreatitis. Activated TRPV1 mediates the release of SP and CGRP from sensory neurons to induce neurogenic inflammation. SP regulates the release of cytokines to induce plasma extravasation and neutrophil infiltration *via* activation of NK-1R. By interacting with calcitonin-like receptor (CLR) and receptor activity modifying protein 1 (RAMP1), CGRP can cause arteriolar vasodilatation. These processes contribute to acute pancreatitis (Grady et al., 2000; Warzecha et al., 2001). TRPV1 can be activated directly by lipoxygenase products, including anandamide, heat, LTB4, and hydrogen ions. Indirect activators of TRPV-1 include ethanol, BAs, proteases, and bradykinin, which are important mediators of pancreatic inflammation (Vigna et al., 2014; Shahid et al., 2015). The related literature has demonstrated that pancreatic vacuole and lysosomal compartments have high proton contents under experimental pancreatitis conditions (Niederau and Grendell, 1988). These acidic compartments, when damaged following pancreatic injury, help lower the tissue pH sufficiently to activate TRPV1 at physiologic temperatures. High proton concentrations can also be produced in tissue ischemia, infection, and inflammation (Bevan and Geppetti, 1994). Shahid et al. (2015) demonstrated that administration of caerulein or intraductal BAs in mice leads to the production of LTB4 by pancreatic acinar cells, which activates TRPV1 on primary sensory nerves to induce AP. Vigna et al. (2011) also showed that common pancreaticobiliary duct obstruction causes an increase in pancreatic LTB4 concentrations that in turn mediates TRPV1 activation, resulting in AP in adult rats. These results support the notion that LTB4 mediates the effects of inflammatory agents on TRPV1 activation. In addition, another study indicated that both TRPV1 and TRPA1 contribute to pancreatic inflammation and pain and provided evidence of synergistic interactions between TRPV1 and TRPA1, which are critical for the development of pancreatitis (Schwartz et al., 2011). Moreover, it has been reported that TRPV1-dependent and TRPA1-dependent neurogenic inflammation is required for the transition from AP to chronic pancreatitis (CP) and pain-related behaviors. Once the transition occurs and CP is established, disease progression occurs independent of the activity of the two channels (Schwartz et al., 2013).

Xu et al. (2007) demonstrated that systemic administration of the TRPV1 antagonist SB-366791 markedly reduced both visceral pain behavior and referred somatic hyperalgesia in rats with CP compared with control animals. They concluded that TRPV1 upregulation and sensitization contribute to hyperalgesia in CP and therefore represent a useful target for treating pancreatic inflammatory pain. This finding suggests that TRPV1 also plays an important role in CP, which is an inflammatory disease primarily affecting the exocrine pancreas that is characterized by irreversible, progressive destruction of the organ, resulting in both severe exocrine and endocrine insufficiency. The destruction of the pancreatic parenchyma is accompanied by local infiltration of inflammatory cells. The main symptom of patients with CP is abdominal pain, which is characterized by severe intermittent or long-lasting and persistent pain. There is evidence to support that the pain caused by CP is due to pancreatic neuropathy. Intrapancreatic nerve damage appears to support the maintenance and deterioration of neuropathic pain (Bockman et al., 1988). The perineurium of intrapancreatic nerves is frequently infiltrated by immune cells in CP. The neurolemmal sheaths of the nerves are damaged and thus are no longer able to provide a protective barrier between the axons and the surrounding connective tissue (Bockman et al., 1988). The nerves become increasingly susceptible to harmful substances and cytokines in the extracellular matrix. SP and CGRP have been shown to be involved in this neurogenic inflammation, which can contribute to local inflammation (Weihe et al., 1991). SP regulates the release of cytokines such as TNFa, IL-1, -2, -6, and -8; increases neutrophil extravasation; regulates macrophage attraction and activation; and causes neutrophil chemotaxis and degranulation of mast cells by activating the G protein-coupled neurokinin receptor NK-1R (Santoni et al., 2000). Clinically, patients with CP and elevated NK-1R mRNA levels develop more intense, more frequent, and more persistent pain. The expression of NK-1R in inflammatory cells of CP also reflects the relationship between SP and inflammatory cells (Shrikhande et al., 2001). NGF increases rapidly in inflamed organs *via* activation of released TNFa, and IL-1b also plays an important role in neurogenic inflammation in CP. NGF can regulate the function of mast cells and macrophages. In addition, NGF can activate TrKA receptors expressed on sensory nerve fibers innervating the site of inflammation (McMahon, 1996). In CP, TRPV1 in intrapancreatic nerves can be activated by intrapancreatic neurotrophic factors, including NGF and artemin, which are overexpressed in pancreatitis (Elitt et al., 2006), resulting in the release of SP and CGRP in the dorsal horn. SP regulates the release of cytokines such as TNFa and IL-1b in inflamed tissues *via* activation of NK-1R. These cytokines interact with TRPV-1 and further increase NGF levels, resulting in a vicious cycle that allows for the maintenance and deterioration of neuropathic pain in CP.

### TRPV1 and Cardiovascular Diseases

#### TRPV1 and Hypertension

Hypertension is a complex disease caused by genetic and environmental interactions that can lead to changes in the function and structure of the heart and cardiovascular system. Hypertension is mainly characterized by a persistent increase in peripheral vascular tone and abnormal vasomotor function, and it is often accompanied by vascular remodeling and increased vascular resistance. Current research has shown that the TRPV 1 channel is heavily involved in sensing blood pressure fluctuations (Hao et al., 2009, 2011; Li et al., 2014). Immunofluorescence results showed that TRPV1 was expressed in the nerve fibers and terminals in the adventitia of the ascending aorta and aortic arch and afferent fibers in the nodule ganglia (Hao et al., 2009). A study by Hao et al. suggests that a long-term high-salt diet leads to endothelium-dependent relaxation of mesenteric resistance and elevation of nocturnal blood pressure in mice. However, high-salt diet-induced endothelial dysfunction and nocturnal hypertension are reduced after long-term use of capsaicin (Hao et al., 2011). Currently, the mechanism by which TRPV1 regulates blood pressure may be related to the following pathways: (1) when TRPV1 is activated in the nerve fibers that control the cardiovascular system, including the heart and systemic blood vessels, it promotes the release of neuropeptide SP and CGRP, which participate in the regulation of peripheral vasoconstriction and reduce the blood volume by diuresis to lower blood pressure (Alawi and Keeble, 2010; Feng and Wang, 2010). (2) Activation of TRPV1 promotes increased phosphorylation of protein kinase A and nitric oxide synthase as well as endothelial cell synthesis and NO release, dilating blood vessels and ultimately reducing blood pressure (Alawi and Keeble, 2010). (3) TRPV1 can be considered an intravascular mechanosensor that can regulate blood pressure by sensing the changes in mechanical pressure (Alawi and Keeble, 2010; Baylie and Brayden, 2011). (4) TRPV1 can be activated when blood pressure increases throughout the body, thereby activating the WNK1 and SGK1 signaling pathways and resulting in decreased expression of the epithelial sodium channel (α-ENaC), which is crucial to the regulation of renal sodium absorption and systemic blood pressure. This effect reduces the reabsorption of Na^+^ and water, ultimately reducing cardiac output and systemic vascular pressure (Li et al., 2014). However, the role of TRPV1 in blood pressure regulation is controversial. Ohanyan et al. (2011) found that TRPV1 increases aortic pressure by enhancing endothelin production and activating endothelin A receptor (ETA). Marshall et al. (2013) examined TRPV1 in a high-fat diet mouse model, and the study results showed that the blood pressure of the wild-type mice in the high-fat diet condition was higher than that in TRPV1-knockout mice. The possible causes for the controversy over the role of TRPV1 in regulating blood pressure may be related to the following factors: (1) there are structurally similar spliced proteins that compete with TPRV1 for binding to ligands and inhibit TRPV1 function after translational modification of TRPV1 (Attila et al., 2014). (2) TRPV1 activation by an agonist can reduce the sensitivity of the aortic baroreceptors and thus suppress decompression reflexes. (3) Smooth muscle cells of the coronary arteries only partially react to agonists, making the antihypertensive effect apparent (Czikora et al., 2012). Therefore, further research is needed to determine the effect of TRPV1 on blood pressure regulation.

#### TRPV1 and Atherosclerosis

Atherosclerosis is a disease characterized by thickening and hardening of the arterial wall, loss of elasticity, and reduction in the lumen. Due to the excessive accumulation of plaque around the arterial wall, blood circulation through these vessels becomes difficult and may eventually lead to the occurrence of cardiovascular disease (Albini et al., 2014). Platelets are thought to play an important role in the pathophysiology of atherosclerosis (Huo and Ley, 2004). Researchers such as Harper AG have found that TRPV1 receptors are present on platelets, which can promote the release of 5-HT and other inflammatory mediators that are beneficial to platelet activation, leading to the formation of atherosclerosis (Harper and Brownlow, 2010). In addition to platelets, the increase in foam cells is also an important event leading to the occurrence of atherosclerotic plaques. The main sources of foam cells are mononuclear macrophages and vascular smooth muscle cells (VSMC) (Rosenfeld and Ross, 1990). Li et al. (2014) provided evidence demonstrating that TRPV1 activation can cause endoplasmic reticulum stress and induce autophagy, which ultimately inhibits the formation of foam cells in VSMCs. Liqun et al. also demonstrated that TRPV1 activation may ameliorate the atherosclerosis caused by a high-fat diet in mice (Liqun et al., 2011). Further studies have found that TRPV1 activation increased the expression of protein kinase A/uncoupling protein 2 (PKA/UCP2) in endothelial cells, ultimately inhibiting mitochondrial dysfunction and attenuating high-fat-diet-induced coronary artery lesions (Xiong et al., 2016). The current mechanisms for TRPV1 agonists to improve atherosclerosis are as follows: (1) Capsaicin, a TRPV1 agonist, regulates lipid metabolism by reducing the accumulation of lipids in cells, indicating that capsaicin may play a role in the prevention of atherosclerosis. Liqun et al. (2011) reported that the number of lipid droplets in VSMCs in the normal group was significantly lower than that in the TRPV 1^−/−^ mice after capsaicin treatment. The activation of the calcineurin and protein kinase A-dependent signaling pathways by calcium ions after activation of TRPV 1 increases the expression of ATP-binding cassette transporter A1 (ABCA1) and decreases the expression of low-density lipoprotein-associated protein-1 (LRP1) to enhance cellular cholesterol efflux and reduce cholesterol influx, ultimately reducing atherosclerosis induced by a high-fat diet in mice. (2) Agonists of TRPV1 promote NO production, which can dilate blood vessels and lower blood pressure, thereby delaying the development of atherosclerosis. Excessive high-fat diet intake can significantly impair coronary vasodilation. However, these effects are extremely exaggerated and are accompanied by increased ROS production and decreased NO production in the endothelium. Supplementation with dietary capsaicin can improve coronary vasodilation induced by a high-fat diet. Scholars have also revealed that capsaicin can promote PKA phosphorylation, reduce mitochondrial ROS production and endothelial mitochondrial dysfunction, and subsequently upregulate UCP2 expression. That is, activation of TRPV1 upregulates the expression of PKA/UCP2 in endothelial cells to reduce free radical production and increase NO release, improves cardiac dysfunction, and prolongs the lifespan of atherosclerotic mice (Xiong et al., 2016). (3) TRPV1 agonists induce the autophagy of smooth muscle cells to engulf lipids, namely, foam cells. A key early event in atherosclerosis is the formation of foam cells, which are cytoplasmic droplets that accumulate cholesterol esters and triglycerides, and autophagy can reduce the accumulation of total cholesterol and cholesterol esters in these cells. Lipid droplets are sent to lysosomes, hydrolyzed to produce free cholesterol, and then secreted (Ouimet et al., 2011).

#### Relationship Between TRPV1 and Myocardial Ischemia, Repair and Reconstruction After Myocardial Infarction

Myocardial ischemia refers to a decrease in blood perfusion of the heart, which results in a decrease in oxygen supply to the heart, abnormal myocardial energy metabolism, and a pathological condition that does not support the normal functioning of the heart. However, when the heart is reperfused with blood, the degree of tissue damage is further aggravated, and organ function is further deteriorated; that is, ischemia-reperfusion injury occurs. Recent studies have found that TRPV1 may be involved in mechanisms that protect against myocardial ischemia-reperfusion injury (Lihong and Wang, 2005; Qin et al., 2008; Zheng et al., 2015). Studies have shown that TRPV1 exerts protective effects on cardiac function in myocardial ischemia-reperfusion injury, in addition to as a molecular receptor for pain (Lihong and Wang, 2005). TRPV1 channels are expressed on the sensory neurons that innervate the myocardium, ventricles of the heart, epicardial surface of the heart, and VSMCs. Lihong and Wang (2005) found that myocardial injury was more pronounced in isolated TRPV1 knockout mice than in wild-type mouse hearts. Administration of a TRPV1 antagonist (chipin) enhanced myocardial damage in isolated wild-type hearts, suggesting that TRPV1 may have a sustained protective effect on ischemia-reperfusion injury after activation. Studies have found that endogenous SP is involved in mediating myocardial protection after persistent ischemia-reperfusion injury. However, postischemic recovery in the mouse heart was significantly inhibited after the administration of capsaicin, which indicates that TRPV1 activation enhances the release of SP to protect the heart when myocardial ischemia occurs. Wei et al. demonstrated that decreased expression of the metabolic syndrome-dependent TRPV1 channel and reduced CGRP release result in greater ischemic reperfusion injury in isolated mouse hearts (Wei et al., 2009). The current research shows that the protective mechanism of TRPV1 on ischemia-reperfusion injury is as follows: (1) low pH or proteases activate protease-activated receptor 2 (PAR2), which belongs to the G protein-coupled receptor family, to stimulate TRPV1 channels *via* PKA/PKC signaling and enhance the release of CGRP and SP in order to provide cardioprotective effects (Zhong and Wang, 2009). (2) Eicosanoids and a low pH may also activate the TRPV1 channel and increase intracellular Ca^2+^ to enhance CGRP and SP release from synaptic vesicles (Qin et al., 2008). CGRP and SP bind to the respective receptors to increase the release of the anti-inflammatory cytokine IL-10, thereby reducing TNF-α levels. Decreased levels of TNF-α can reduce ROS and neutrophil infiltration to attenuate ischemia-reperfusion injury in heart tissues (Lei et al., 2016). (3) Myocardial ischemia results in the release of metabolites such as bradykinin, which activates the TRPV1 receptor in sympathetic afferent fibers to evoke a sympathoexcitatory reflex, thereby transmitting a defense signal for chest pain (Zahner et al., 2003).

Repair after myocardial infarction is a dynamic process that begins with acute inflammation, followed by granulation tissue formation and extracellular matrix deposition. Repair and reconstruction processes overlap each other during this period (Dewald et al., 2005). Transforming growth factor (TGF-β1) is one of the most critical elements of fibrosis and may be the main factor regulating infarct repair. Activated TGF-1 can induce sustained biological effects through downstream Smads after myocardial infarction (Hao et al., 1999). Han et al. (2008) showed that TRPV1 plays a protective role in promoting damage repair after myocardial infarction. Deletion of TRPV1 may result in increased expression of matrix metalloproteinase-2 (MMP-2) and vascular endothelial growth factor. Increased expression of MMP-2, which degrades the extracellular matrix, causes ventricular dilatation and thinning of the ventricular wall and thereby decreases cardiac function. Overexpression of vascular endothelial growth factor produces abnormal vascular proliferation, aggravates ventricular remodeling and deterioration of cardiac function, and ultimately inhibits repair after myocardial infarction. In addition to describing the beneficial effects of TRPV 1 channel activation, a study reported that activation of these channels may also be detrimental. Wang et al. (2014) examined several indicators of cardiac remodeling and function in rats with chronic heart failure (CHF) treated with resin toxin (RTX). Their data show a reduction in chamber size, lower left ventricle end-diastolic pressure, reduced cardiac hypertrophy, and attenuated cardiac fibrosis and apoptosis. Their results demonstrate that selectively deleting TRPV1-expressing cardiac sympathetic afferent reflex afferents elicits protective effects against deleterious cardiac remodeling and autonomic dysfunction in CHF. The reason why their findings are contrary to the results of other researchers may be related to the following factors: RTX not only deletes TRPV1 receptors in cardiac afferents but also damages the nerve endings that express TRPV1 rather than blocking only TRPV1 receptors. The cardiac sympathetic afferent terminals expressing TRPV1 also express many other sensory receptors, such as tachykinins and purinergic receptors. RTX also impairs these sensory receptors following damage to TRPV1-expressing cardiac afferent nerve endings (Zahner et al., 2003). The existence of these factors may lead to the emergence of other results.

Scientists have used various pharmacological agents, including N-oleoyl dopamine, capsaicin, resin toxin, CGRP8-37, RP-67580, capsazepine, and TRPV1 knockout mice (Zhong and Wang, 2008; Cao et al., 2014), to explore the involvement of TRPV1 channels and subsequent CGRP and SP release in ischemia-reperfusion injury and myocardial remodeling. The results indicate that activation of the TRPV1 channel can modulate cardiovascular responses and that these channels can be a promising target for the treatment of cardiovascular disease.

### TRPV1 and Respiratory Diseases

Respiratory diseases affect the quality of life of people worldwide. Many researchers have devoted their careers to the study of the pathogenesis of respiratory diseases, including efforts to determine which ion channels are involved in diseases such as asthma, chronic obstructive pulmonary disease (COPD), and chronic cough (Caterina et al., 1997; Jordt and Ehrlich, 2007; Zhu et al., 2009). It has been found that the function and/or expression of TRP channels may be altered in the context of respiratory diseases, including asthma, COPD, and chronic cough respiratory diseases. In this section, we outline the pathophysiological role of TRPV1 in chronic airway diseases such as asthma and COPD.

#### TRPV1 and Airway Inflammation

TRPV1 is widely distributed in the sensory nerve fibers of the respiratory system, especially in cells with C- and Aδ-type fibers, and is functionally identified as a nociceptor. In the respiratory system, TRPV1 can be activated by a variety of endogenous and exogenous ligands, including capsaicin, resiniferatoxin, and PGE2 citric acid, with low pH. Activation of TRPV1 expressed in sensory neurons leads to an increase in the intracellular Ca^2+^ concentration and, subsequently, the excitability of cells, thereby promoting the secretion of tachykinin and CGRP in nerve endings, which act on respiratory effector cells such as cholinergic neurons, mucinous gland cells, inflammatory cells, trachea, and VSMCs, resulting in bronchoconstriction, tracheal mucosal edema, inflammatory cell chemotaxis, protein secretion, and other physiological functions (Brozmanova et al., 2012). Therefore, it is currently believed that airway inflammation caused by TRPV1 is mainly “neurogenic inflammation.” In a recent work by Irving H Zucker and his group (Shanks et al., 2018), their data suggested that the pulmonary reflex observed in vagotomized animals is due to signaling through a spinal sympathetic afferent pathway rather than either vagal afferents or through a systemic circulatory effect. They also found that activation of chemosensitive pulmonary spinal afferent nerve fibers by bradykinin and capsaicin can evoke an increase in renal sympathetic nerve activity, mean arterial pressure, and heart rate. These data also confirm that the reflex is mediated by TRPV1 sensory afferents, suggesting the presence of TRPV1 in the excitatory pulmonary chemosensitive sympathetic afferent reflex. This finding may have important clinical implications in pulmonary conditions that induce sensory nerve activation, such as pulmonary inflammation and the inhalation of chemical stimuli. In addition, a potential influence of activation of pulmonary spinal afferents expressing TRPV1 is their contribution to the increased risk of cardiovascular events and a poor prognosis in patients with existing disease. For instance, those suffering from COPD are more likely to have major adverse cardiovascular events from cardiovascular comorbidities than those without COPD (Villar Alvarez et al., 2008; Ooi et al., 2018).

It is also important to note the presence and function of TRPV1 in nonneural cells in the airways, including airway epithelium in the upper and lower respiratory tract, smooth muscle cells, fibroblasts, and T cells (Song and Morice, 2017). Several studies have shown that TRPV1 activation stimulates the release of proinflammatory cytokines from bronchial epithelial cells, including tumor necrosis factor-α, prostaglandin E2, NGF and interleukins (ILs), to promote airway inflammation (Sadofsky et al., 2012; Zholos, 2015). Functional TRPV1 protein has been confirmed in cultured primary bronchial epithelial cells by patch-clamp experiments, and the TRPV1 agonist capsaicin induces dose-dependent release of IL-8, which can be blocked by the antagonist capsazepine (McGarvey et al., 2014). TRPV1 mRNA expression was increased in whole lung tissues from COPD patients compared with those of healthy nonsmokers (Baxter et al., 2014). Moreover, it is overexpressed in the airway epithelium and submucosa of asthmatic patients (McGarvey et al., 2014). These studies indicate that increased TRPV1 expression is associated with the pathophysiology of the disease in both neuropathic and nonneuronal cell types. Investigators have proposed that environmental prototype particulate matter (PM) is sensed by TRPV1 and have shown that RPV1 mediates the induction of several important proinflammatory cytokine/chemokine genes in lung epithelial cells in response to PM (Deering-Rice et al., 2012). Chronic exposure to cigarette smoke (CS) is a key factor in the development of COPD and is thought to cause an inflammatory response in the airway that is characterized by high levels of leukocyte infiltration and the release of inflammatory mediators that drive disease progression and decrease lung function. In a study by Baxter et al., TRPV1 was shown to play a role in CS-induced extracellular ATP release, which facilitates the production of IL-1β and IL-18 (Baxter et al., 2014). These findings provide novel insights into the role of these channels in the generation of PM-induced and CS-induced airway inflammation. Bertin et al. (2014) found that functional TRPV1 is expressed in mouse CD4^+^T cells, which are activated upon stimulation of the T cell antigen receptor (TCR), contributing to Ca^2+^ influx and TCR signaling and resulting in T cell activation. These results indicate that TRPV1 plays an important role in the activation of CD4^+^T cells and the acquisition of inflammatory properties. TRPV1 channels were identified in human monocyte-derived dendritic cells (DCs), the most efficient antigen presenting cells (APCs), which were overexpressed during cytokine-induced *in vitro* differentiation. The TRPV1 channel appears to be involved in the inhibition of DC maturation and functions, such as bacterial phagocytosis and cytokine secretion (Toth et al., 2009). In contrast, in mouse bone marrow-derived DCs, TRPV1 channel activation induces cell maturation and activation (Basu and Srivastava, 2005). It has also been evidenced that the TRPV1 channel may be involved in the physiology and pathophysiology of inflammation and the immune system. In fact, TRPV1 channels expressed in immune and inflammatory cells mediate different, sometimes opposite, reactions that are currently difficult to assign to specific, coordinated pathways and processes. Therefore, further research is needed to elucidate the signaling mechanism of TRPV1 in modulating immune inflammatory responses, which will contribute to clinical research on the development of novel therapies for inflammatory and immune diseases as well as the appropriate selection of TRPV1 channel agonists and antagonists.

#### TRPV1 and Cough

Cough is one of the most common symptoms of respiratory diseases, which seriously affects the quality of life of patients. Acute cough (under 8 weeks duration) is usually the result of a viral or bacterial upper respiratory tract infection and is often resolved after the infection has cleared (Irwin et al., 2006). Chronic cough can last for more than 8 weeks and is associated with a variety of respiratory diseases, such as asthma, COPD, and idiopathic pulmonary fibrosis (IPF). These patients can be triggered by innocuous stimuli that lead to uncontrolled cough (Pratter, 2006). When the airway sensory nerve is subjected to changes in the physical and chemical environment and reaches a certain threshold, an action potential is generated that is transmitted from the vagus nerve to the different regions of the medulla, where the reflex is processed. The signal then causes reflex bronchoconstriction through the sympathetic efferent signal or a cough caused by the motor nerve to the larynx, ventilator, and diaphragm. These reflexes are usually protective, but airway reflexes become abnormal in the disease, leading to increased cough symptoms (Pacheco, 2014). The role of various TRP channels, including TRPV4, TRPA1, TRPA1, and TRPV1, in the cough reflex has been known. However, current research has revealed little about the expression of TRPV4 in peripheral sensory neurons that innervate the lungs. Evidence to date suggests that TRPM8 may play a minimal role in airway physiology, with conflicting results reported (Grace et al., 2014). Studies have demonstrated the important role of TRPA1 in the lung defense system (Grace et al., 2012). However, the TRPV1 channel is widely distributed in the respiratory system and has been functionally identified as a nociceptor. Low levels of TRPV1 in the airways indicate its role in airway defense, and studies have shown the role of TRPV1 sensory nerves in normal cough reflexes. Cough induced by substances that activate TRPV1, such as capsaicin and citric acid, in human and animal models is inhibited by TRPV1 antagonists (Grace et al., 2014). Another study has shown that SB705498 ligand, a TRPV1 antagonist, can reduce the sensitivity of cough reflexes to capsaicin (Zhang et al., 2018). Increased expression of TRPV1 in sensory nerves has been demonstrated in patients with chronic cough (Mitchell et al., 2005). Moreover, patients with chronic cough have an increase in sensitivity to capsaicin (Pecova et al., 2008). An increased level of bradykinin is shown in the airways of asthmatic patients, which causes cough after activation of TRPV1 (Grace et al., 2012).

Clinical studies have shown that inhaled corticosteroid or cyclooxygenase 2 inhibitors in asthma patients can decrease the sensitivity of patients to capsaicin (Ishiura et al., 2009; Ekstrand et al., 2011). Human rhinovirus, which is the main cause of the common cold, can contribute to asthma exacerbations. A recent study has shown that rhinovirus has the ability to infect nerve cells, and the infection can lead to an increase in expression of TRPV1 (Abdullah et al., 2014). In addition, another study revealed the relationship between TRPV1 single nucleotide polymorphisms (SNPs) and the protective effects against the presence of wheezing in a group of asthmatic patients (Cantero-Recasens et al., 2010). Several SNPs of TRPV1 have also been shown to be associated with cough symptoms in non-asthmatic patients and increased sensitivity to cough caused by environmental stimuli or smoking (Smit et al., 2012). Patients with COPD also showed increased ATP expression, which causes cough after TRPV1 activation, suggesting that it is possible to increase the release of inflammatory mediators induced by increased levels of ATP, which acts on the TRPV1 channel to enhance the cough response. The current mechanism for TRPV1 leading to cough may be the following: (1) TRPV1 acts as the main cough receptor, and when it is activated by temperature, capsaicin, H^+^, inflammatory mediators, etc., it mediates the rapid influx of calcium ions and sodium, resulting in the depolarization of neurons and the generation of nerve impulses. Subsequent activation of C-type fibers releases neuropeptides such as SP and CGRP to induce neurogenic inflammation and increase cough sensitivity (Undem and Carr, 2010). (2) Released SP and CGRP can also induce tissue edema and airway smooth muscle contraction, indirectly activating retinoic acid receptors (RARs) to elicit a cough (Arash et al., 2013). (3) The release of inflammatory mediators induced by TRPV1 activation may be a factor in enhancement of the cough reflex. The excess mucus secreted by effector cells after TRPV1 activation in COPD airways may also cause chronic cough through mechanical stimulation (Hogg, 2004).

#### Relationship Between TRPV1 and Airway Hyperresponsiveness

Airway hyperresponsiveness (AHR) refers to the highly sensitive state of the airway to various stimulating factors, such as allergens, physical and chemical factors, exercise, drugs, etc., which is manifested by an excessive or premature contraction response of the airway when the patient is exposed to these stimuli. AHR is an important factor in the development of asthma. It is generally believed that chronic inflammation of the airway is one of the important mechanisms leading to AHR. When the airway is affected by allergens or other stimuli, a variety of inflammatory cells release inflammatory mediators and cytokines, airway epithelial damage, subepithelial nerve endings, etc., resulting in high airway reactivity. AHR not only occurs in individuals with asthma but also in long-term smokers, individuals with viral upper respiratory tract infections, those with COPD, etc., but the degree of AHR is relatively mild. Although some scholars have ruled out the role of TRPV1 in AHR (Raemdonck et al., 2012), many current results show that TRPV1 plays a role in the development of AHR. Rehman et al. found that TRPV1 inhibitors attenuate AHR and airway remodeling changes in IL-3-induced asthma models (Rehman et al., 2013). The results of their study suggested that TRPV1 plays a vital role in AHR and airway remodeling. Another study using a guinea pig model also showed that the use of TRPV1 inhibitors can reduce airway hyperresponsiveness (Delescluse et al., 2012). In addition, it has been reported that inhibition of the function of TRPV1 can attenuate the response of signaling pathways and alleviate the symptoms of children with chronic asthma (Cantero-Recasens et al., 2010). A recent study also showed that TRPV1 is involved in the development of AHR in response to the chemical sensitizer toluene-2,4-diisocyanate (TDI) (Devos et al., 2016). Numerous studies have shown that the TRPV1 channel located on the airway sensory nerve is activated when subjected to physical or chemical stimulation, leading to the release of CGRP and bradykinin, which acts on effector cells, and thereby activating proinflammatory mediators, followed by airway inflammation and, ultimately, AHR and airway remodeling. At the same time, TRPV1 can be activated by various pro-inflammatory mediators, including lipoxygenase (LOX) metabolites, which creates a positive feedback effect. It has also been confirmed that 15-LOX deficiency either by pharmacological compounds or knockout can alleviate asthma characteristics (Hajek et al., 2008). These findings suggest that alleviation of asthma features by inhibiting the production of 15-LOX may also occur through the reduction in TRPV1 activation.

The role of TRPV1 in pain and cough has been well studied, and it is considered an attractive therapeutic target for pain- and cough-related diseases (McLeod et al., 2008). Several pharmaceutical companies have adopted various therapeutic modalities that have been investigated in animal studies and human clinical trials (Chen et al., 2011). SB705498, a potent selective TRPV1 antagonist, can exert significant antitussive effects and reduce neurogenic inflammation by reducing the expression of potent TRPV1 antagonism, demonstrating superior efficacy and potency in SP and CGRP in a capsaicin-induced cough model of guinea pigs (Zhang et al., 2018). Belvisi et al. also confirmed that XEN-D0501 preclinical and clinical capsaicin challenge studies, which significantly reduced capsaicin-induced cough (Belvisi et al., 2017). Researchers have also injected microcapsules of capsaicin into the commissural nucleus of the solitary nucleus (NTS) of rats to induce a bradypnoeic response, with a tendency toward apnea, but tidal volume was not affected (Geraghty et al., 2011). In the latest study, the application of capsaicin in the medulla had a transient inhibitory effect on the respiratory center and subsequent excitatory effects on the respiratory rhythm and periodical augmentation of the inspiratory burst pattern (Tani et al., 2017). This research provides a theoretical basis for developing new ideas and new therapeutic targets for controlling respiratory disorders. All of these studies have shown that the TRPV1 channel is of great importance as a potential therapeutic target for the treatment of respiratory diseases.

## Conclusions

Undoubtedly, as research on TRPV1 continues, the possibility of TRPV1 becoming a therapeutic target for the treatment of diseases is advanced. Among all TRP channels, TRPV1 antagonists are the most studied in drug development and clinical trials. The mechanisms of action of TRPV1 in the digestive system, respiratory system, and cardiovascular system are continuously being revealed. However, there are still mechanisms that are not fully clarified and are even controversial. However, regardless of the TRPV1 distribution throughout the body, the role of TRPV1 is mainly due to its direct or indirect activation by various ligands, causing intracellular calcium elevation, triggering action potentials, and transmitting nociceptive signals to the nerve center. It can also activate a series of signaling pathways in the cell, resulting in extensive cellular responses to promote the regulation of corresponding physiological and pathological functions. The widespread distribution of TRPV1 throughout the body also represents a challenge, as adverse reactions and off-target effects are a real threat when TRPV1 is used as a therapeutic target. The possibility of adverse unintended effects has limited the development of the use of TRPV1 antagonists. For instance, TRPV1 functions as a thermosensor for hot temperatures. Patients showed significant and long-lasting increases in body temperature after administration of the TRPV1 antagonist AMG517 (Gavva et al., 2008) in Phase 1 and Phase 1b clinical trials and MK-2295 (Xia et al., 2011) in a phase 2A POC trial. These trials were terminated due to safety concerns before its analgesic effect could be determined. Another study on the TRPV1 antagonist AZD1386 for pain relief was discontinued due to elevated liver enzymes. At present, most of the research on TRPV1 is basic science research; only a few studies have clinical relevancy, and research on TRPV1 has mainly focused on chronic pain, while fewer studies have been conducted on its role in other diseases, such as ulcers, hypertension, and some pathological processes, such as airway remodeling and myocardial ischemia. In summary, further research is needed on the structure, characteristics and physiological functions of TRPV1 and its changes in pathological conditions.

## Author Contributions

CC and XY contributed to the conception of this review and completed figures drawing. QD and QL analyzed literatures and wrote the manuscript. RX and JX revised the manuscript.

### Conflict of Interest Statement

The authors declare that the research was conducted in the absence of any commercial or financial relationships that could be construed as a potential conflict of interest.
